# Evaluation of nosocomial infections through contact patterns in a small animal hospital using social network analysis and genotyping techniques

**DOI:** 10.1038/s41598-021-81301-9

**Published:** 2021-01-18

**Authors:** Amara Churak, Chaithep Poolkhet, Yutaka Tamura, Tomomi Sato, Akira Fukuda, Sukanya Thongratsakul

**Affiliations:** 1 Section of Epidemiology, Department of Veterinary Public Health, Faculty of Veterinary Medicine, Kasetsart University, Kamphaeng Saen Campus, Nakhon Pathom, 73140 Thailand; 2 Laboratory of Food Microbiology and Food Safety, Division of Health and Environmental Science, School of Veterinary Medicine, Rakuno Gakuen University, Ebetsu, Hokkaido 069-8501 Japan

**Keywords:** Clinical microbiology, Health services, Epidemiology

## Abstract

Nosocomial infections or hospital-acquired infections (HAIs) are common health problems affecting patients in human and animal hospitals. Herein, we hypothesised that HAIs could be spread through human and animal movement, contact with veterinary medical supplies, equipment, or instruments. We used a combination of social network analysis and genotyping techniques to find key players (or key nodes) and spread patterns using *Escherichia coli* as a marker. This study was implemented in the critical care unit, outpatient department, operation room, and ward of a small animal hospital. We conducted an observational study used for key player determination (or key node identification), then observed the selected key nodes twice with a one-month interval. Next, surface swabs of key nodes and their connecting nodes were analysed using bacterial identification, matrix-assisted laser desorption/ionisation-time of flight mass spectrometry, and pulsed-field gel electrophoresis. Altogether, our results showed that veterinarians were key players in this contact network in all departments. We found two predominant similarity clusters; dendrogram results suggested *E. coli* isolates from different time points and places to be closely related, providing evidence of HAI circulation within and across hospital departments. This study could aid in limiting the spread of HAIs in veterinary and human hospitals.

## Introduction

Nosocomial infections or hospital-acquired infections (HAIs) are an important problem in both human and animal hospital and health care services^1^. These infections cause further harm to their health beyond the condition for which they were initially admitted. In veterinary medicine, studies have reported many different kinds of pathogens that cause HAIs in small animal hospitals, such as *Cryptosporidium*^2^, *Acinetobacter baumannii*, *Enterococcus faecalis*, *Enterococcus faecium*, *Staphylococcus intermedius*^3^, *Staphylococcus aureus*^4^, and *Escherichia coli*^5–7^. In addition, *E. coli* and *Clostridium difficile* containing a pool of antimicrobial resistance genes have been recovered from environmental sites of these hospitals^8^. Studies on the potential spread of HAI pathogens in small animal hospital environments are necessary to improve our understanding of this issue, and social network analysis (SNA) is an effective tool for this kind of investigation. In general, SNA has two major components: nodes and ties. Nodes are units of interest that are ties. Ties are a relation or connection of a pair or a group of nodes in a studied network.

SNA has been used to understand contact patterns of nosocomial infections in hospitals and healthcare centres. A previous study reported methicillin-resistant *S. aureus* (MRSA) colonisation in infants and concluded that patients were at risk of MRSA infection from siblings and people with whom they shared nursing care^9^. In a study in Japan, researchers noticed that physicians spread nosocomial pathogens between different hospital wards^10^. Using a simulation, researchers also reported that HAIs can be transmitted between patients. In this case, they suggested isolating the infected patient, increasing hand sanitizing, and vaccinating staff to help limit the spread of nosocomial infections^11^.

Kasetsart University Veterinary Teaching Hospital (KUVTH) at Bang Khen is the largest small animal hospital in Thailand. According to our data, more than 500 animals received services at this hospital daily in 2015. This hospital thus has a high density of people and animals, indicative of a high contact pattern during hospitalization, revealing that our patients could contract an HAI easily through contact during their activities in the hospital. This study hypothesised that a contact pattern could be maintained through human and animal movement, as well as contact with veterinary medical supplies, equipment or instruments. The results of this study could be useful for both human and animal hospital management.

## Results

### Key player determination

In total, 49 animals of 49 owners were used within the study period. We collected data from 39 (39/49; 79.59%) dogs, 9 (9/49; 18.37%) cats, and 1 (1/49; 2.04%) rabbit. In the outpatient department (OPD), critical care unit (CCU), ward, and operation room (OR), there were 22 (22/49; 44.90%), 20 (20/49; 40.82%), 4 (4/49; 8.16%), and 3 (3/49; 6.12%) animals, respectively. In the OPD, there were 16 (16/49; 32.65%), 5 (5/49; 10.20%), and 1 (1/49; 2.04%) dog, cat, and rabbit owners, respectively. In the CCU, there were 17 (17/49; 34.69%) dog owners and 3 (3/49; 6.12%) cat owners. In the ward, there were 3 (3/49; 6.12%) dog owners and 1 (1/49; 2.04%) cat owner. Three (3/49; 6.12%) dogs received our services in the OR.

**Figure 1:**
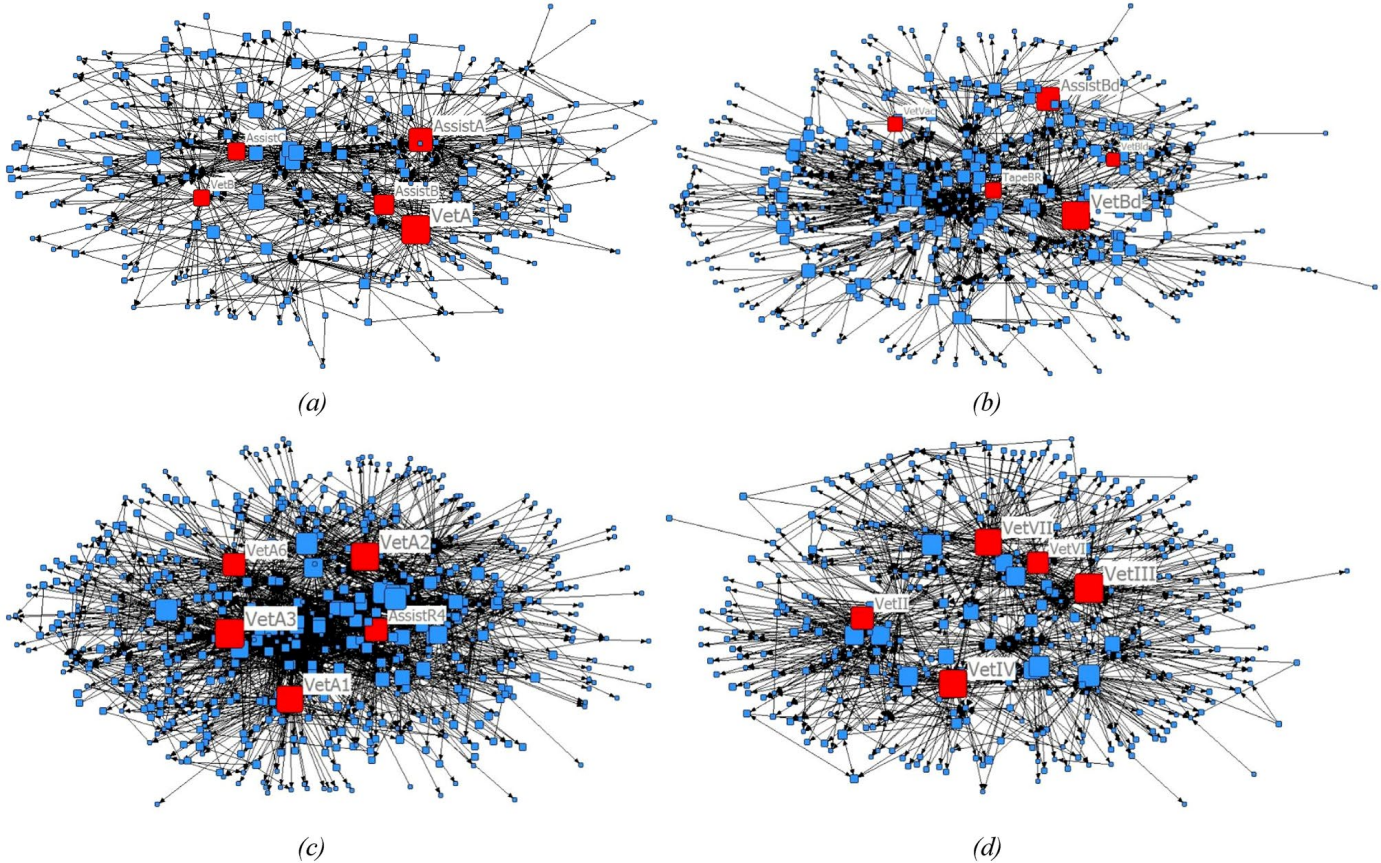
Sociogram of contact networks categorised by department. Each square symbol represents an individual node. The node size varies based on its key player determination value. The red square node represents the five nodes with the highest key player determination values. **(a)** Critical care unit; **(b)** outpatient department; **(c)** operation room; **(d)** ward. The figure was generated in NetDraw 2.160 (https://sites.google.com/site/netdrawsoftware/home).

Table 1 and Fig. 1 show the sociograms of the CCU, OPD, OR, and ward. The five highest values of key player determination in each department were predominantly the human type. Veterinarians had the highest key player values in all departments, followed by veterinary assistants.

**Table 1:** Nodes with the highest key player determination values per department.

Department	Node ID	Node type	Key player determination value at 0.5 alpha
**Critical care unit (no. of nodes = 258, no. of ties = 851)**			
	VetA	Human	48.031
	AssistA	Human	39.438
	AssistB	Human	31.125
	AssistC	Human	25.250
	VetB	Human	23.906
**Outer patient department (no. of nodes = 465, no. of ties = 1366)**			
	VetBd	Human	27.250
	AssistBd	Human	21.438
	TapeBd	Supply	13.625
	VetVacc	Human	12.250
	VetBld	Human	11.563
**Operation room (no. of nodes = 545, no. of ties = 2276)**			
	VetA3	Human	59.625
	VetA2	Human	57.250
	VetA1	Human	54.500
	AssistR4	Human	46.000
	VetA6	Human	43.422
**Ward (no. of nodes = 391, no. of ties = 1134)**			
	VetIII	Human	39.014
	VetIV	Human	37.195
	VetVII	Human	35.289
	VetII	Human	29.059
	VetVI	Human	28.014

### Contact networks of key players

**Figure 2:**
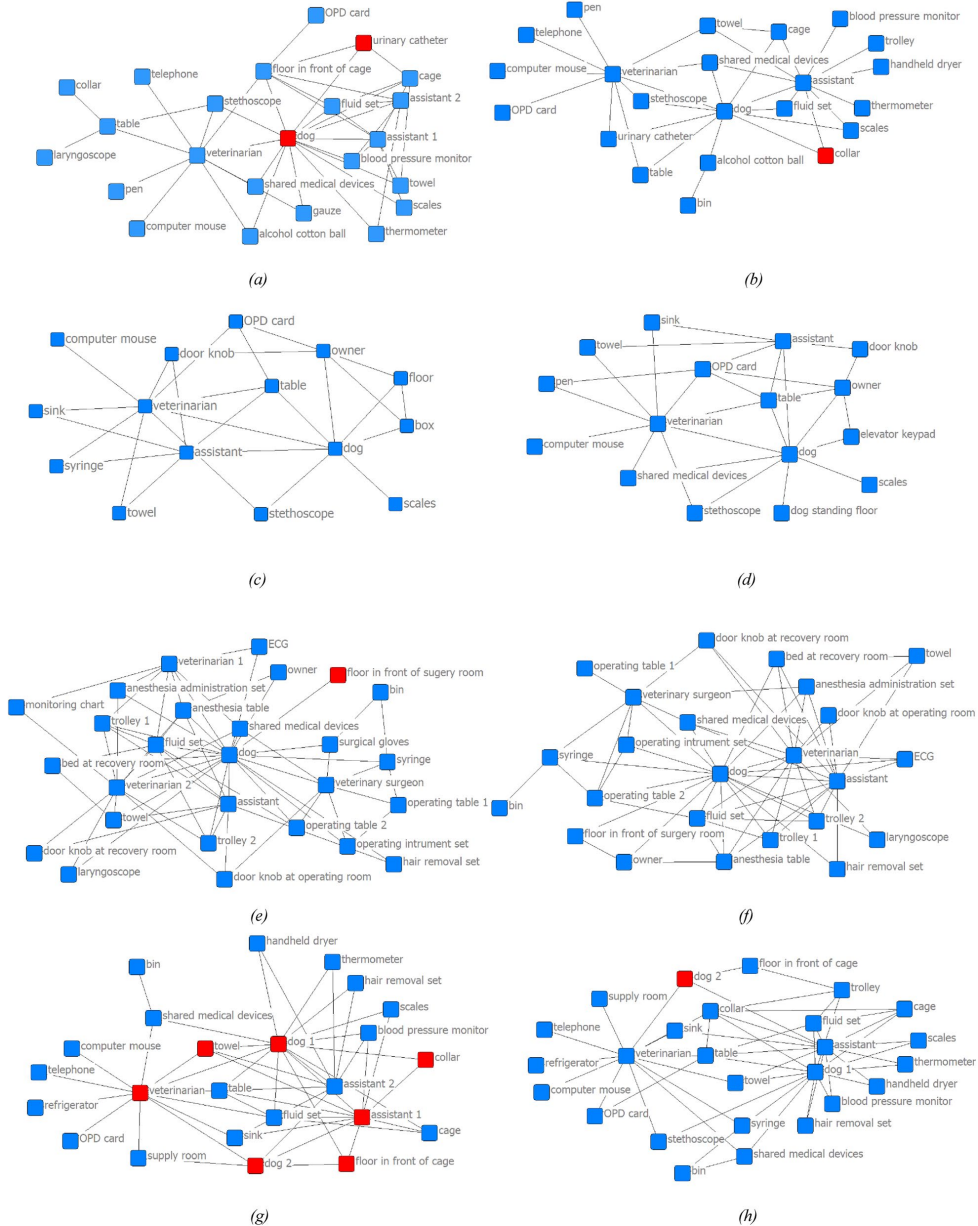
Sociogram of observed networks with surface swabs categorised by department and time of sampling. Red nodes represent positive *E. coli* samples. **(a)** Critical care unit: first sampling event (23 nodes, 44 ties); **(b)** critical care unit: second sampling event (22 nodes, 32 ties); **(c)** outpatient department: first sampling (15 nodes, 27 ties); **(d)** outpatient department: second sampling event (16 nodes, 29 ties); **(e)** operation room: first sampling event (27 nodes, 66 ties); **(f)** operation room: second sampling event (24 nodes, 60 ties); **(g)** ward: first sampling event (24 nodes, 53 ties); **(h)** ward: second sampling (26 nodes, 52 ties). The figure was generated in NetDraw 2.160 (https://sites.google.com/site/netdrawsoftware/home). https://sites.google.com/site/netdrawsoftware/home.

Based on the results obtained in the first phase of our study, we concluded that veterinarians were key players in the contact network of all departments. Figure 2 shows that veterinarians contacted veterinary assistants, animals, and supplies in rooms of different departments. It appeared that the OR and ward had higher ties, with a higher number of nodes, than the CCU and OPD, reflecting more connections made by veterinarians in these areas.

We initially collected sample swabs of 23, 15, 27, and 24 nodes in the CCU, OPD, OR, and ward, respectively. One month later, we swabbed 22, 16, 24, and 26 nodes in the CCU, OPD, OR, and ward, respectively. We used more than one swab when nodes had large surface areas, resulting in a total of 51, 23, 60, and 60 swabs used in the CCU, OPD, OR, and ward, respectively, for the first sampling and 45, 33, 53, and 52, respectively, in the second sampling event. A total of 377 swabs were used for sample collection in this study.

### *E. coli* identification and genotyping

After analysing the bacterial culture, matrix-assisted laser desorption/ionisation-time of flight mass spectrometry (MALDI-TOF MS), and pulsed-field gel electrophoresis (PFGE) results, we found that 18 (18/377; 4.77%) *E. coli* isolates were detected among the two sampling times and the four evaluated departments. Regarding the ward, nine (9/18; 50%) and one (1/18; 5.56%) positive samples were obtained in the first and second sampling times, respectively. In the CCU, five (5/18; 27.78%) and two (2/18; 11.11%) positive samples were obtained in the two sampling times, while only one (1/18; 5.56%) positive sample was obtained in the OR at the first sampling event. We found no positive samples from the OPD.

**Figure 3:**
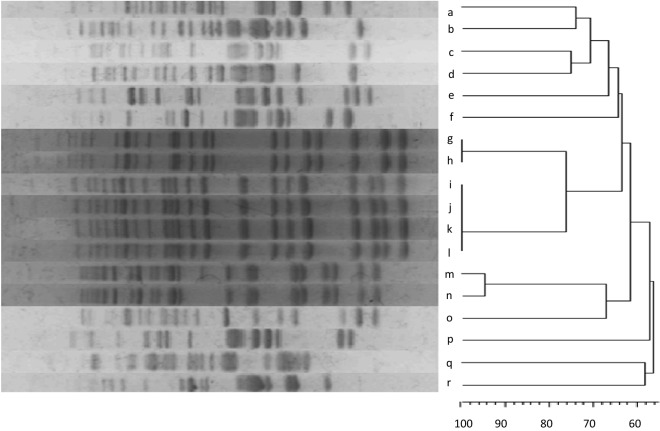
Dendrogram of the results of pulse field gel electrophoresis (PFGE) of all 18 *E. coli* isolates from each department at both sampling times. **(a)** Isolation from the skin of dog no. 2 at the second sampling event; **(b)** isolation from a multipurpose towel in the ward at the first sampling event; **(c)** isolation from the skin of dog no. 1 in the ward at the first sampling event; **(d)** isolation from the skin of dog no. 1 in the ward at the first sampling event; **(e)** isolation from an assistant’s cover shirt in the ward at the first sampling event; **(f)** isolation from a veterinarian’s hand in the ward at the first sampling event; **(g)** isolation from the skin around a dog’s wound in the critical care unit (CCU) at the first sampling event; **(h)** isolation from a collar in the ward at the second sampling event; **(i)** isolation from a urinary catheter in the CCU at the first sampling event; **(j)** isolation from the skin of a dog in the CCU at the first sampling event; **(k)** isolation from a collar in CCU at the second sampling event; **(l)** isolation from a floor section where dogs stood in front of the operation room (OR) at the first sampling event; **(m)** isolation from a urinary catheter in the CCU at the first sampling event; **(n)** isolation from a urinary catheter in the CCU at the first sampling event; **(o)** isolation from the floor in front of a cage in the ward at the first sampling event; **(p)** isolation from the floor in front of a cage in the ward at the first sampling event; **(q)** isolation from the skin of a dog in the ward at the first sampling event; **(r)** isolation from a collar in the ward at the first sampling event. The figure was generated in BioNumerics 6 (https://www.applied-maths.com/bionumerics).

As shown in Figs. 2 and 3, among samples from the first sampling event in the ward, we found *E. coli* on the floor in front of a cage (2), the skin of dog no. 1 (2), a multipurpose towel (1), a veterinary assistant’s shirt (1), a veterinarian’s hand (1), the skin of dog no. 2 (1), and on a collar (1). During the second sampling in the ward, we found a positive sample from the skin of dog no. 2 (that presented a positive sample during the first sampling event). In the CCU, we found *E. coli* on a urinary catheter (3), the skin of a dog’s wound (1), and on a healthy skin area of the same dog (1) during initial sampling. During the second sampling, we found two positive *E. coli* samples from different sides of the same collar. Another *E. coli*-positive sample was collected in the first event of a section of the floor where dogs stood in front of the OR.

Figure 3 presents the dendrogram that indicates two predominant clusters of similarities. The first cluster comprehended *E. coli* detected on the skin of a dog’s wound from the initial samples collected in the CCU and on a collar from the second samples collected in the CCU. Another cluster consisted of *E. coli* from a catheter and the skin of a dog that was detected in the first sampling event in the CCU, from a collar detected the second sampling in the CCU, and from a section of the floor in front of the OR, detected in the first sampling event. In addition, we found a cluster of *E. coli* in the first sample of a towel from the ward and in the second sample of dog no. 2 from the ward with a moderately close relationship.

## Discussion

In this study, SNA and genotyping techniques were used to study nosocomial infections in a small animal hospital. The genotyping profile of *E. coli* was used to evaluate the spread of HAIs, and we found that humans such as veterinarians may play a key role in this phenomenon. Moreover, the dendrogram showed that isolates obtained from different times and places displayed clustering of *E. coli*. This provides evidence that HAIs can circulate within and spread across hospital departments.

In this study, results indicated that veterinarians play an important role in the contact networks of the studied hospital departments. This is due to the fact that veterinarians are at the core of the work activities of the hospital. These professionals must keep in mind that they have a higher chance of coming into direct contact with contaminated people, animals, or objects than others; therefore, personal hygiene restriction on limiting the spread of HAIs is necessary. In addition, veterinary assistants can also spread nosocomial pathogens through contact and should be aware of their role in reducing the spread of HAIs. A previous study reported that limiting physicians’ movements between wards effectively reduced the epidemic size of a nosocomial infection in a human hospital^10^. Recently, Gröschel et al. reported that a human-associated strain of *Stenotrophomonas maltophilia* was predominantly present, which indicates that humans could be a source of HAIs by direct or indirect transmission^18^. Thus, restricting human activities in hospitals as well as enforcing regulations on personnel sanitizing could limit the spread of HAIs. For example, the staff should avoid walking across hospital departments or one-way paths should be designed in the hospital for staff on duty.

In general, the prolonged stay of a dog in the ward might increase the number of positive *E. coli* samples obtained in this department. In this study, we found *E. coli* in the ward on both sampling times, as well as a cluster of *E. coli* among these samples. In general, our hospital cleaning protocol in ward for all supplies, instruments, and equipment undergo a daily cleaning and sanitizing program using a disinfectant which contain potassium peroxymonosulfate and sodium chloride as active ingredients. For the dirty floor, sodium hypochlorite was often used for cleaning and disinfection. For the cages, hot steam under pressure was used as an additional cleaning method. For staff, self-sanitization both in the hospital and their homes were practiced. However, some of the animals in the ward are not cleaned daily, and HAI pathogens could therefore survive for many days or even months. Nocera et al. observed that a prolonged hospital stay resulted in *A. baumannii* colonisation in a dog^19^. Restrictive practices to minimise infection, as well as personal hygiene and sterilisation techniques, could interrupt this chain and limit HAIs. Moreover, handling more than one animal at the same time should be avoided by veterinary professionals to prevent HAI exposure between animals.

We found similar *E. coli* strains among the CCU swabs collected with a one-month interval. This indicates that daily cleaning and sanitizing might not be enough to remove all possible HAIs and that the bacteria were possibly circulating in the hospital. The hospital mainly used chemical disinfection, similar to the ward, for cleaning and sanitizing the environment, and we suggest that protocols should be re-evaluated. Furthermore, more modern procedures should be adopted, such as pulsed xenon ultraviolet disinfection; this method has been reported as an additional disinfection method for reducing aerobic bacteria and MRSA in a hospital environment^20^. On the other hand, the results of our study showed that dog skin is also a possible source of HAIs, and therefore limiting animal movement, cleaning dogs when possible, and using disposable supplies with each patient could reduce the potential for HAI spread. Moreover, the development of a surveillance program for early HAI identification in veterinary practices is required^21^.

Detection of similar *E. coli* strains between the CCU and OR indicated that HAI pathogens can spread between different departments. Based on the results of the first phase of our study, we hypothesised that *E. coli* might be moved to/from the CCU and OR by human contamination. Considering that our sampling procedures were conducted in different weeks between the CCU and OR, this also confirmed that *E. coli* could circulate in a hospital environment. Different hospitals might have different HAI spread patterns, and this study can provide information for a hospital administrator to make an informed decision to control and limit HAI spread in their hospital.

A limitation of this study was that we used only one environmental bacterium as a marker for understanding the HAI spread patterns, which could cause our results to be underestimated. However, the use of SNA made our study more specific to the key player (i.e. the veterinarians), enhancing the evidence of HAI spread in animal hospitals as described above. We highly recommend that future studies combine SNA and molecular techniques to further understand HAI patterns in hospitals. Moreover, our observational process might have affected the behaviour of the hospital staff and owners. However, our study involved a veterinary teaching hospital where most of the staff and owners are familiar with this kind of environment.

This study showed evidence of HAI circulation in a small animal hospital using *E. coli* as a marker. Using SNA and genotyping analysis, we found possible evidence of HAI spread within and between departments. These results increase our understanding of the patterns of nosocomial pathogen spread, and can be applied to reduce HAIs in both animal and human hospitals.

## Methods

All animal owners were asked for permission to observe them and their animal’s activities in KUVTH until the end of their stay at the animal hospital. Thereafter, informed verbal consent was applied prior to the observation. Our methodology was approved by Kasetsart University’s Institutional Animal Care and Use Committee (KU-IACUC) for consideration of ethics in animal usage (Approval no. ACKU59-VET-040). All procedures involving participants were performed in accordance with the ethical standards of KU-IACUC and the principles of the Declaration of Helsinki.

A brief description of the study area: KUVTH is veterinary teaching hospital of the main campus of Kasetsart University which is located in Bangkok, Thailand. The hospital contains 29 units/departments. Most of the units are veterinary medical services covering a range of internal medicine, surgery, ward, radiology, emergency, rehabilitation, blood bank, exotic pets, necropsy, and unit of veterinary specialists. The hospital has usable area of approximately 30,000-square-meter with 140 veterinarians and approximately 300 other employees.

### Study design

This study was conducted in KUVTH in Bang Khen, Bangkok, Thailand from January to December 2015. Four important departments that had previously been associated with nosocomial infections were selected for evaluating HAI patterns: the CCU, OPD, OR, and ward. Inclusion criteria for the selected departments were: (1) CCU, a department with an expressive HAI rate^12^; (2) OPD, a department with a high density of animal patients; (3) OR, a department with a high level of disinfecting cleaner use; and (4) ward, a department with prolonged patient stays.

**Figure 4:**
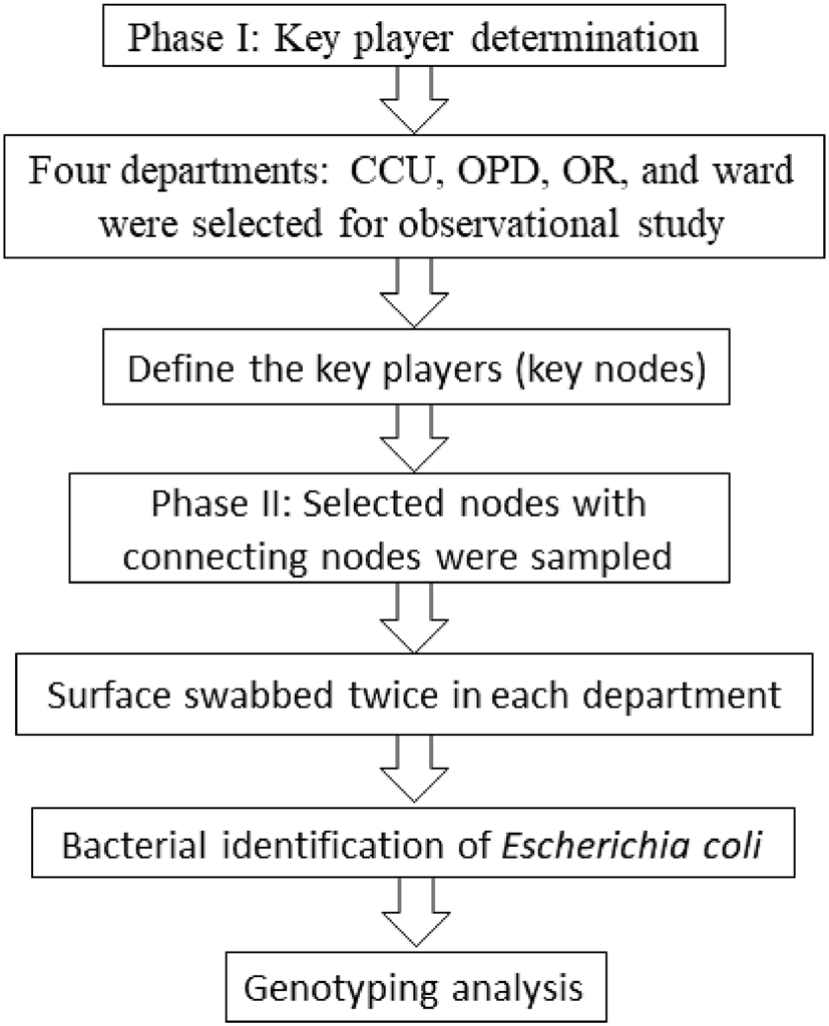
Flow chart of the research study. *CCU* critical care unit, *OPD* outpatient department, *OR* operation room.

We divided our study into two phases: the first phase was an observational study used for key player determination^13,14^. In this phase, the units of interest (nodes) were individual humans, animals, equipment, and instruments. Each node was identified by physical separation. The second phase used the results obtained in the first phase. Key players or selected nodes were observed twice for each department with a one-month interval between observations. We sampled the nodes using surface swabs for bacterial identification and genotyping. We used *E. coli* as the target bacterium for genotype analysis^5,12,15^ in order to investigate HAI spread. In this phase, the connected nodes (people, animals, and objects that possibly exposed the key players) of key players were observed and swabbed as much as possible (Fig. 4).

### Observational study and key player determination

In the first phase of this study, data was collected through purposive sampling. At the beginning of the day, only the animal owners were allowed in the hospital reception area, and we observed all activities until the owners were finished. All nodes contacted by the owners and their animals were recorded with directions. Animal identification and appearance were used to avoid duplicate data in case the owners brought the animal back to the hospital. The data were continuously collected every week (2 to 3 days per week) until no new nodes were found. All data and key player metrics were analysed in NetDraw 2.160 (Analytic Technologies, Lexington, Kentucky, USA) using key player determination at 0.5 alpha attenuation (Table 1). Moreover, a directed values sociogram was produced (Fig. 1).

### Bacterial identification and genotyping

In the second phase of this study, the surfaces of selected nodes with connecting nodes in the CCU, OPD, OR, and ward were swabbed for bacterial identification and genotyping analysis. The surface of each node was swabbed using one to eight swabs depending on the surface area or its high-contact sites (for example, a human hand, the back of an animal, a door knob, the floor where animals stand, and the handing bar of a trolley). In each department, we collected samples twice with a one-month interval using the same selected nodes from the first phase of the study. In addition, we only swabbed one department per week. Three hours at the beginning of the day were used for collecting samples. In total, three hundred and seventy-seven samples were collected in this study.

All the samples were kept in a cooling box at 4 °C before being sent to a laboratory within 24 h. Briefly, the samples were cultured on EC broth (Merck, Darmstadt, Germany) and were incubated for 24 h at 37 ± 0.5 °C. Next, the samples were cultured on MacConkey agar and Coliform agar for *E. coli*, and three typical colonies were chosen for identification using a biochemical IMViC test (indole, methyl red, Voges-Proskauer, citrate test; Bioxon, Mexico City, Mexico)^16^. All *E. coli* samples were preserved at − 20 °C in tryptic soy broth (TSB) (Difco, Detroit, USA) with 30% glycerol (Scharlau, Barcelona, Spain) for molecular approach. MALDI-TOF MS (Bruker, Bremen, Germany) was used to confirm the identification of *E. coli* strains. Finally, PFGE was performed for genotyping analysis of DNA fingerprinting as previously described^17^. Briefly, genomic DNA was digested using *Xbal* and PFGE was performed on a CHEF DR III (Bio-Rad, USA) for 19 h at 11.3 °C with an initial forward time of 2.2 s, a final switch time pf 54.2 s, and voltage of 6.0 V. PFGE profiles and cluster analysis were identified using BioNumerics 6 (Applied Maths, Austin, Texas, USA). The results were used to evaluate the dendrogram similarities of the isolates (Fig. 3).

## Data Availability

The datasets generated during and/or analysed during the current study are available from the corresponding author on reasonable request.
